# Automated Emergent Large Vessel Occlusion Detection Using Viz.ai Software and Its Impact on Stroke Workflow Metrics and Patient Outcomes in Stroke Centers: A Systematic Review and Meta-analysis

**DOI:** 10.1007/s12975-025-01354-0

**Published:** 2025-05-08

**Authors:** Khalid Sarhan, Ahmed Y. Azzam, Mostafa Hossam El Din Moawad, Ibrahim Serag, Abdallah Abbas, Ahmed E. Sarhan

**Affiliations:** 1 Faculty of Medicine, Mansoura University, Mansoura, Egypt; 2 Faculty of Medicine, October 6 University, Giza, Egypt; 3 Alexandria Main University Hospital, Alexandria, Egypt; 4 Faculty of Medicine, Suez Canal University, Ismailia, Egypt; 5 Faculty of Medicine, Al-Azhar University, Damietta, Egypt; 6 Lecturer of Neurology, Al-Azhar University, Cairo, Egypt; 7 Medical Research Group of Egypt (MRGE), Negida Academy, Arlington, MA USA

**Keywords:** Machine learning, Artificial intelligence, Large vessel occlusion, Stroke workflow metrics

## Abstract

The implementation of artificial intelligence (AI), particularly Viz.ai software in stroke care, has emerged as a promising tool to enhance the detection of large vessel occlusion (LVO) and to improve stroke workflow metrics and patient outcomes. The aim of this systematic review and meta-analysis is to evaluate the impact of Viz.ai on stroke workflow efficiency in hospitals and on patients’ outcomes. Following the PRISMA guidelines, we conducted a comprehensive search on electronic databases, including PubMed, Web of Science, and Scopus databases, to obtain relevant studies until 25 October 2024. Our primary outcomes were door-to-groin puncture (DTG) time, CT scan-to-start of endovascular treatment (EVT) time, CT scan-to-recanalization time, and door-in–door-out time. Secondary outcomes included symptomatic intracranial hemorrhage (ICH), any ICH, mortality, mRS score < 2 at 90 days, and length of hospital stay. A total of 12 studies involving 15,595 patients were included in our analysis. The pooled analysis demonstrated that the implementation of the Viz.ai algorithm was associated with lesser CT scan to EVT time (SMD −0.71, 95% CI [−0.98, −0.44], *p* < 0.001) and DTG time (SMD −0.50, 95% CI [−0.66, −0.35], *p* < 0.001) as well as CT to recanalization time (SMD −0.55, 95% CI [−0.76, −0.33], *p* < 0.001). Additionally, patients in the post-AI group had significantly lower door−in door-out time than the pre-AI group (SMD −0.49, 95% CI [−0.71, −0.28], *p* < 0.001). Despite the workflow metrics improvement, our analysis did not reveal statistically significant differences in patient clinical outcomes (*p* > 0.05). Our results suggest that the integration of the Viz.ai platform in stroke care holds significant potential for reducing EVT delays in patients with LVO and optimizing stroke flow metrics in comprehensive stroke centers. Further studies are required to validate its efficacy in improving clinical outcomes in patients with LVO.

**Supplementary Information:**

The online version contains supplementary material available at 10.1007/s12975-025-01354-0.

## Introduction

Acute ischemic stroke (AIS) remains a primary cause of long-term disability and mortality in the USA and worldwide, despite substantial progress in stroke management over the past 30 years [1]. Large vascular occlusion (LVO) accounts for roughly 30–40% of ischemic strokes [2, 3], resulting in 60% of post-stroke dependency and mortality at 90 days, and 90% of post-stroke mortality at 6 months [4]. The primary standard of therapy for patients with AIS within 4.5 h of symptom onset continues to be intravenous thrombolysis [5, 6]. Nonetheless, this initial line of treatment has a low use rate due to its limited therapeutic time window and suboptimal recanalization rate for LVO [7].

Mechanical thrombectomy (MT) has been demonstrated to be both effective and safe for treating cerebral LVO, enhancing patients’ clinical outcomes according to the findings of randomized clinical trials (RCTs) [8, 9]. The duration between symptom onset and MT is crucial to patient outcomes, as delays in administering MT can markedly diminish the likelihood of favorable results [10]. Current care systems exhibit fundamental difficulties, particularly regarding the transfer between primary and comprehensive (thrombectomy-capable) stroke centers, as well as the prolonged procedures that ensue upon the patient’s arrival at the comprehensive stroke center (CSC) [11, 12].

Prior reports suggest that the factors impeding MT are multifaceted and frequently influenced by delays in the diagnosis of LVO strokes and communication with neurointerventionalists [13–18]. Minimizing treatment delivery time is essential for ensuring the level of care for patients with LVO. Implementing suitable workflow modifications in imaging is a crucial intervention to minimize delays. Alterations in workflow resulted in substantial decreases in the time required for thrombectomy. Alterations to the computed tomography (CT) workflow encompassed a “no turn back” policy from the CT scan suite to the angiography suite, as well as reconfiguring the process to enable imaging and treatment within the same room, all of which enhanced the time to thrombectomy. Furthermore, the significance of sophisticated imaging modalities (CT perfusion and CT angiography) must be acknowledged in this context. The application of improved imaging techniques, especially within the expanded time frame (6–24 h), may delay thrombectomy for qualifying patients, but does not adversely affect functional outcomes. Consequently, improved imaging modalities are a crucial component of management in this group [19, 20].

**Fig. 1:**
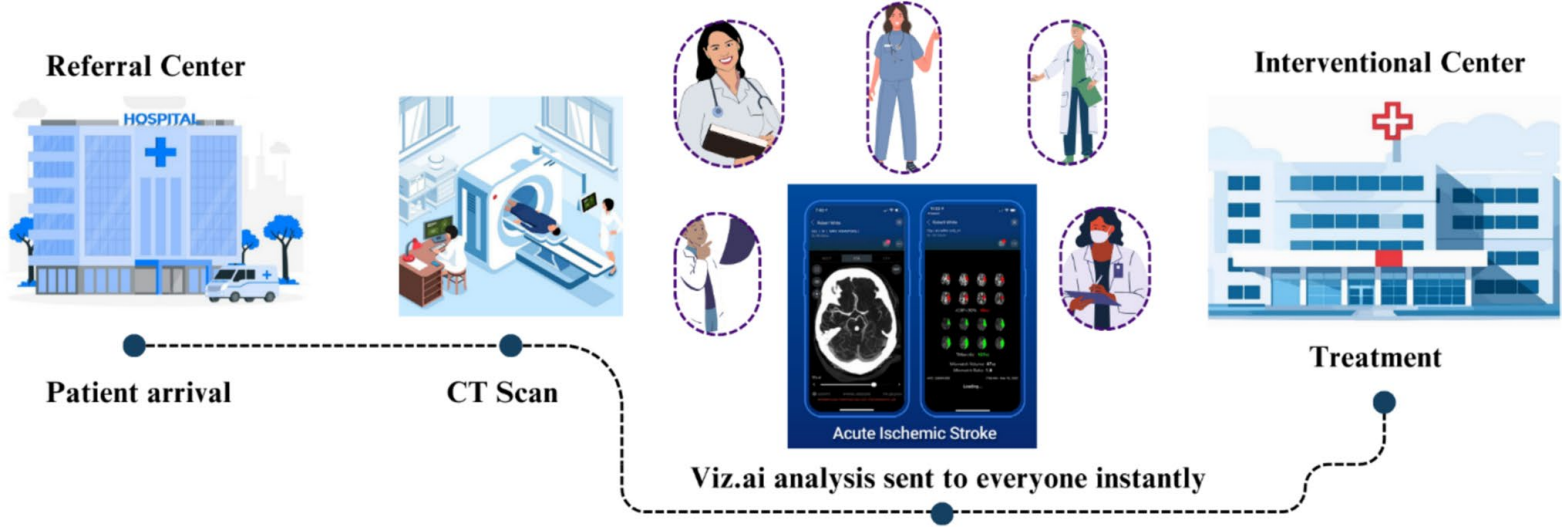
Workflow of LVO detection and transfer using Viz.ai software

To reduce delays in the management of LVO stroke patients undergoing MT, artificial intelligence (AI)-driven mobile stroke systems are being implemented globally. These platforms are claimed to enhance stroke workflow by integrating AI-driven automated LVO identification, real-time mobile high-resolution neuroimaging file sharing, and multi-user communication functionalities [21, 22]. The most often employed platforms are Viz.ai and RapidAI. It is important to recognize that Viz.ai is a comprehensive software platform that integrates artificial intelligence with workflow optimization tools. While the deep learning algorithm for large vessel occlusion (LVO) detection is a key feature, the overall impact on stroke workflow metrics is likely multifactorial. The platform also includes automated alerts, real-time image sharing, and secure mobile communication, all of which enhance coordination among stroke team members. Therefore, improvements in door-to-intervention times should be attributed not only to the AI-based detection algorithm but also to the broader efficiencies introduced by modern communication technology (Fig. 1). Numerous hospitals have indicated enhancements in stroke workflow following the introduction of these systems [23–27]. Conclusions drawn from these previous reports should be regarded with caution for three reasons. Initially, these investigations have predominantly been smaller, single-center research, which raises concerns over their reproducibility. Secondly, the metrics examined in previous studies have been intricate and significantly affected by numerous variables beyond the AI platform’s control (e.g., spoke hospital door-in–door-out times, time-to-groin puncture, duration of stay, and patient outcomes). Finally, the majority of research has been serial cohort studies that evaluated metrics before and following the adoption of AI-based stroke platforms, frequently with significant intervals between cohorts, during which various other modifications in stroke workflow presumably occurred [24]. Therefore, we conducted the current systematic review and meta-analysis to investigate the efficacy of the use of Viz.ai software on the workflow parameters and clinical outcomes of stroke patients with LVO undergoing MT. Pooling the results from all the available literature can produce a strong evidence and more robust results.

## Methods

Adhering to the Cochrane Handbook of Systematic Reviews of Interventions at each step [28] and following the Preferred Reporting Items for Systematic Reviews and Meta-Analyses (PRISMA) statement’s guidelines, we conducted this systematic review and meta-analysis [29].

### Database Searching

Using the following search terms “machine learning” OR “artificial intelligence” OR “deep learning” AND “large vessel occlusion” OR “ischemic stroke,” we searched PubMed, Web of Science, and Scopus for eligible articles that should undergo the screening process to determine their ability to be included in our study. This search was done from inception until October 2024.

### Screening

After database searching, we removed the duplicates from the resulting articles using EndNote version 7 [30] software; then we uploaded the remaining articles on Rayyan software to conduct the process of screening. First, two authors who worked independently conducted the screening by title and abstract to see the eligibility for inclusion, and then they conducted full-text screening of the included articles from the previous step. Any conflicts were solved by consensus or referred to a senior author to resolve.

### Inclusion and Exclusion Criteria

The predetermined inclusion and exclusion criteria used for screening were any cohort studies, retrospective studies, or RCTs that included (1) patients presenting to stroke centers with suspected LVO undergoing CTA, (2) pre and post implementation of the Viz.ai platform in stroke centers for automated evaluation, and (3) studies measuring stroke workflow metrics and patient clinical outcomes. We excluded case reports, reviews, and studies not comparing the pre- and post-implementation of the Viz.ai platform in stroke centers.

### Quality Assessment

For the included observational cohort studies, we used the New Castle Ottawa scale tool provided by Cochrane for the assessment of quality. It is composed of eight questions, with a maximum of one star for each, except for the comparability question that can receive two stars. Therefore, the highest score is 9, while the lowest score is 0. Studies scoring from 0 to 3 were considered of low quality, 4–6 were of moderate quality, and 7–9 were of high quality [31]. The Cochrane risk-of-bias instrument (RoB 2.0) [32], comprising five domains with associated sets of questions, was utilized to assess the risk of bias in RCTs. These domains include randomization, deviations from intended interventions, outcome measurement, missing outcome data, and outcome selection of the reported result. A study can only be considered to have an overall low risk of bias if each of the five domains is classified as low risk individually. If any domain expresses some concerns, then the study is considered to have some problems. Nonetheless, if any domain is judged to be at high risk or if multiple domains raise some red flags, the study is labeled as having a high risk of bias.

### Data Extraction and Outcome Measures

Using Microsoft Excel sheets, two independent authors conducted the process of data extraction to extract the baseline data (setting and stroke centers, duration, study design, sample size, age, gender, and the mean National Institutes of Health Stroke Scale) in addition to the stroke workflow metrics outcomes including: CTA-to-EVT start time, door-to-groin puncture (DTG) time, CTA-to-recanalization time, door-in–door-out, door-to-NIR notification time, rate of EVT post-transfer to comprehensive stroke center (CSC) as well as patient clinical outcomes including symptomatic intracranial hemorrhage (ICH), any ICH, mortality rate, mRS score ≤ 2 at 90 days, and length of hospital stay.

### Statistical Analysis

Using Review Manager version 5.4 software [33], we conducted the meta-analysis of the included studies by pooling the event and total of dichotomous variables to measure the risk ratio (RR) and the mean difference (MD) or standardized mean difference (SMD) for continuous variables at confidence intervals (CI) of 95% and a *p*-value of 0.05. The *I*^2^ was used for testing the heterogeneity, with the *p*-value for significance. To ensure the robustness of our results, we conducted a sensitivity analysis in multiple scenarios, excluding one study in each scenario and evaluating its effect on the overall effect estimate. Using funnel plots, publication bias was visually investigated. The Egger test [34] and Begg-Mazumdar test [35] were used to quantify any additional bias that was observed.

## Results

### Search and Screening Process

**Fig. 2:**
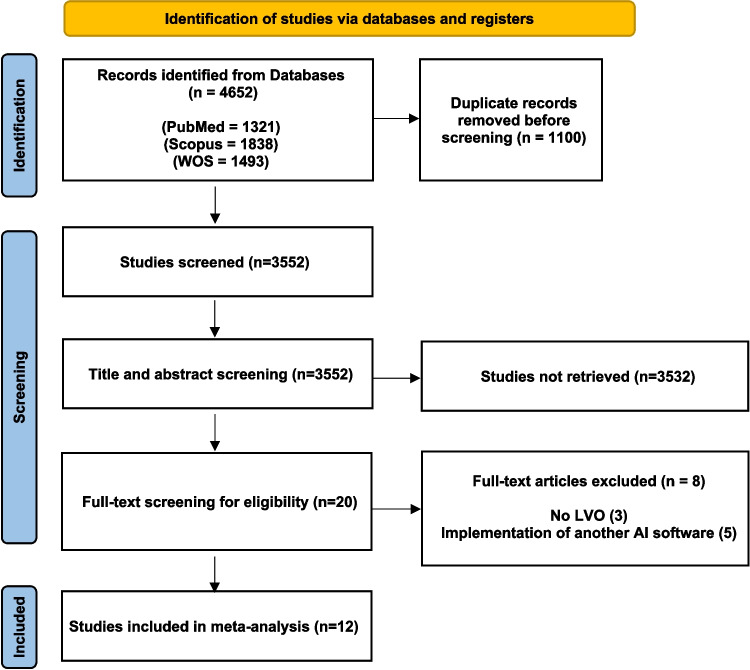
Prisma flow diagram of the included studies

Our search retrieved 4652 studies. Following title and abstract screening, 20 studies were eligible for full-text screening (Fig. 2). A total of 12 studies, including 15,595 patients, met the eligibility criteria and were included in this meta-analysis.

### Baseline Characteristics

The studies involved a total of 6335 patients in the pre-study groups and 9260 patients in the post-study groups, as shown in Table 1. The overall average age was approximately 68.4 years in the pre-study groups and 68.9 years in the post-study groups. Among studies reporting gender, there were 2933 males in the pre-study groups, accounting for about 51.5% of these groups, while the post-study groups included 3346 males, comprising approximately 50.4% of these participants. Refer to Table 1 for further details on patient demographics across the studies.

**Table 1:** Summary and baseline characteristics table

Study ID	Setting and stroke centers	Duration	Design	N° of patients		Male:female		Mean age ± SD, years		Mean NIHSS ± SD	
				Pre	Post	Pre	Post	Pre	Post	Pre	Post
Bonner 2024 [36]	Comprehensive stroke center (5 spokes, 1 hub), Cooper University Hospital, Camden, NJ, USA	December 2018 to August 2022	Retrospective cohort	162	127	75:87	68:59	67.67 ± 18.7	68.53 ± 13.95	-	-
Devlin 2024 [24]	166 facilities (17 states), USA	December 2021 to March 2022	Retrospective cohort	5559	8557	2598:2961	3933:4624	65.49 ± 15.94	66.82 ± 16.30	2.67 ± 2.97	2.33 ± 3.71
Le 2024 [37]	7 PSCs in the greater Houston-area, Houston, TX, USA	January 2021 to February 2022	Retrospective cohort	80	35	38:42	18:17	63.83 ± 16.61	70.33 ± 12.37	8.33 ± 11.32	10.67 ± 10.82
Frost 2024 [38]	Comprehensive stroke network (8 spokes, 1 hub), Cooper University Hospital, Camden, NJ, USA	September 2019 to November 2020	Retrospective cohort	58	74	29:29	40:34	70.33 ± 11.4	68.67 ± 12.85	12 ± 11.4	13.67 ± 10.59
Martinez-Gutierrez 2023 [39]	4 CSCs in the greater Houston-area, Houston, TX, USA	January 2021 to February 2022	RCT	140	103	67:73	54:49	68.67 ± 14.61	69 ± 16.54	17 ± 8.99	16 ± 7.52
Orden 2023 [40]	CSC in University of California, San Diego, San Diego, California, USA	June 2020 to June 2022	Retrospective cohort—quality improvement	47	103	-	-	-	-	-	-
Matsoukas 2023 [41]	3 CSCs and 7 PSCs in The Mount Sinai Health System, New York, New York, NY, USA	January 2020 to December 2021	Retrospective cohort	40	38	19:21	17:21	Median (IQR) 71.5 (17.3)	Median (IQR) 70 (21.8)	15.5 ± 8.5	13.3 ± 8.7
Figurelle 2023 [27]	CSC in University of California, San Diego, San Diego, California, USA	June 2020 to June 2021	Retrospective cohort	47	35	-	-	-	-	-	-
Hassan 2022 [26]	CSC in Valley Baptist Medical Center, Harlingen, TX, USA	November 2016 to May 2020	Retrospective cohort	86	102	51:35	58:44	68.53 ± 13.13	69.87 ± 15.75	16.13 ± 8.33	15.91 ± 7.10
Elijovich 2022 [25]	One hub and two spoke hospitals, Baptist Memorial Hospital Memphis, Memphis, TN, USA	May to December 2019	Retrospective cohort	59	45	27:32	16:29	71 ± 17.48	69.67 ± 15.32	14.33 ± 9.12	11 ± 12.25
Morey 2021 [23]	CSC, 3 TSCs, and 2 PSCs, Mount Sinai Health System, New York, New York, NY, USA	July 2018 to March 2020	Retrospective cohort	29	26	14:15	13:13	76.2 ± 13.9	72.8 ± 15.4	Median (IQR) 17 (7)	Median (IQR) 14 (9.5)
Hassan 2020 [42]	CSC in Valley Baptist Medical Center, Harlingen, TX, USA	February 2017 to May 2019	Retrospective cohort	28	15	15:13	6:9	71.64 ± 12.28	69.13 ± 13.29	18.25 ± 7.43	14.07 ± 6.75

*CSC* Comprehensive Stroke Center, *PSC* Primary Stroke Center, *TSC* Thrombectomy-capable Stroke Center, *RCT* randomized controlled trial, *N°* number, *NIHSS* National Institutes of Health Stroke Scale, *SD* standard deviation, *IQR* interquartile range

### Risk of Bias Assessment and Publication Bias

The 11 observational studies were evaluated using the NOS tool, with nine studies achieving a score of 8 out of 9, indicating *good quality*. Two studies scored 7 out of 9, also reflecting a *good quality* rating. Additionally, one RCT was assessed using the ROB 2 tool, revealing *some concerns* overall in its risk of bias assessment. Overall, these studies demonstrated a generally good quality in terms of bias and methodological rigor (Table 2). Using funnel plots, no significant publication bias was observed for the stroke workflow metrics outcomes (eAppendix 2 Supplementary).

**Table 2:** Risk of bias assessment of the observational studies and RCTs

Study	Risk of bias assessment of the observational studies according to NOS									
	Selection				Comparability	Outcome			Total score	Quality
	Representativeness of the exposed cohort	Selection of the non-exposed cohort	Ascertainment of exposure	The outcome of interest was not present at the start of the study		Assessment of outcome	Was follow-up long enough for outcomes to occur	Adequacy of follow-up of cohorts		
Bonner 2024	⭐	⭐	⭐	⭐	⭐	⭐	⭐	⭐	**8 out of 9**	**Good**
Devlin 2024	⭐	⭐	⭐	⭐	⭐	⭐	⭐	⭐	**8 out of 9**	**Good**
Le 2024	⭐	⭐	⭐	⭐	⭐⭐	⭐	⭐	⭐	**9 out of 9**	**Good**
Frost 2024	⭐	⭐	⭐	⭐	⭐	⭐	⭐	⭐	**8 out of 9**	**Good**
Orden 2023	⭐	⭐	⭐	⭐	⭐	⭐	⭐	⭐	**8 out of 9**	**Good**
Matsoukas 2023	⭐	⭐		⭐	⭐⭐	⭐	⭐		**7 out of 9**	**Good**
Figurelle 2023	⭐	⭐	⭐	⭐	⭐	⭐	⭐	⭐	**8 out of 9**	**Good**
Hassan 2022	⭐	⭐	⭐	⭐	⭐⭐	⭐	⭐	⭐	**9 out of 9**	**Good**
Elijovich 2022	⭐	⭐	⭐	⭐	⭐	⭐	⭐	⭐	**8 out of 9**	**Good**
Morey 2021	⭐	⭐	⭐	⭐	⭐	⭐	⭐		**7 out of 9**	**Good**
Hassan 2020	⭐	⭐	⭐	⭐	⭐⭐	⭐	⭐	⭐	**9 out of 9**	**Good**
	**Risk of bias assessment of the RCT using ROB 2**									
Study	**D1**	**D2**	**D3**	**D4**	**D5**	**Overall**				
Martinez-Gutierrez 2023	Some concerns	Low risk	Low risk	Low risk	Low risk	**Some concerns**				

*D1* bias arising from the randomization process, *D2* bias arising from deviations from intended interventions, *D3* bias arising from missing outcome data, *D4* bias arising from the measurement of outcomes, *D5* bias arising from the selection of the reported result

### Results of Stroke Workflow Metrics Outcomes

**Fig. 3:**
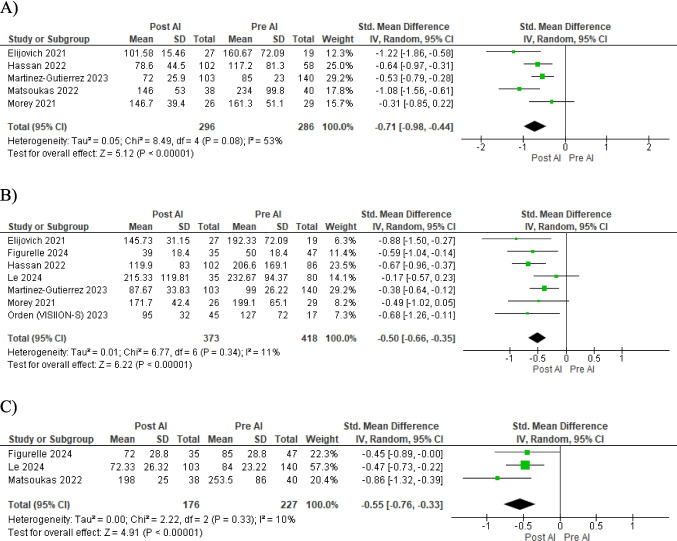

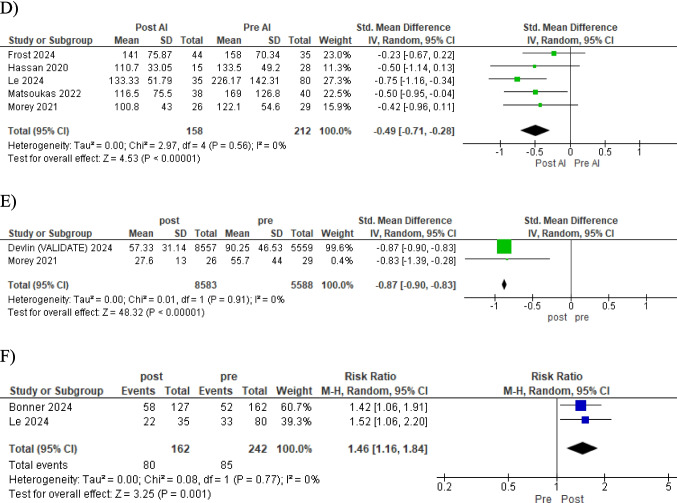
Forest plots of stroke workflow metrics outcomes comparing pre and post-implementation of Viz.ai software. **A** CTA-to-EVT start time, **B** door-to-groin puncture (DTG) time, **C** CTA-to-recanalization time, **D** door-in door-out, **E** door-to-NIR notification time, **F** rate of EVT post-transfer to comprehensive stroke center (CSC)

The most notable finding was a substantial reduction in the time from CT angiography (CTA) to the initiation of endovascular treatment (EVT). The standardized mean difference (SMD) of −0.71 (95% CI −0.98 to −0.44, *p* < 0.00001) indicates a marked acceleration in this crucial phase of stroke management (Figure 3A). This improvement was complemented by a significant decrease in door-to-groin puncture (DTG) time (SMD −0.50, 95% CI −0.66 to −0.35, *p* < 0.00001), suggesting enhanced efficiency in patient preparation and transfer to the angiography suite Figure 3B.

The interval from CTA to successful recanalization was notably shortened (SMD −0.55, 95% CI −0.76 to −0.33, *p* < 0.00001) (Figure 3C) implying more rapid reperfusion of blood flow to ischemic brain regions. In the context of inter-hospital transfers, we observed a significant reduction in door-in–door-out times (SMD −0.49, 95% CI −0.71 to −0.28, *p* < 0.00001). This improvement could be attributed to more efficient triage and decision-making processes facilitated by the VIZ.ai platform (Figure 3D).

Notably, the time to neurointerventional radiologist (NIR) notification was significantly reduced (SMD −0.87, 95% CI −0.90 to −0.83, *p* < 0.00001), potentially contributing to the overall workflow optimization (Figure 3E). Interestingly, the rate of EVT post-transfer to comprehensive stroke centers (CSCs) increased significantly (RR 1.46, 95% CI 1.16 to 1.84, *p* = 0.001) (Figure 3F). To ensure the robustness of our results, we did sensitivity analysis excluding each study at a time and evaluating its impact on the overall effect size. The results remained consistent across different assumptions, supporting the robustness of our results (eAppendix 1 Supplementary).

### Results of Clinical Outcomes

**Fig. 4:**
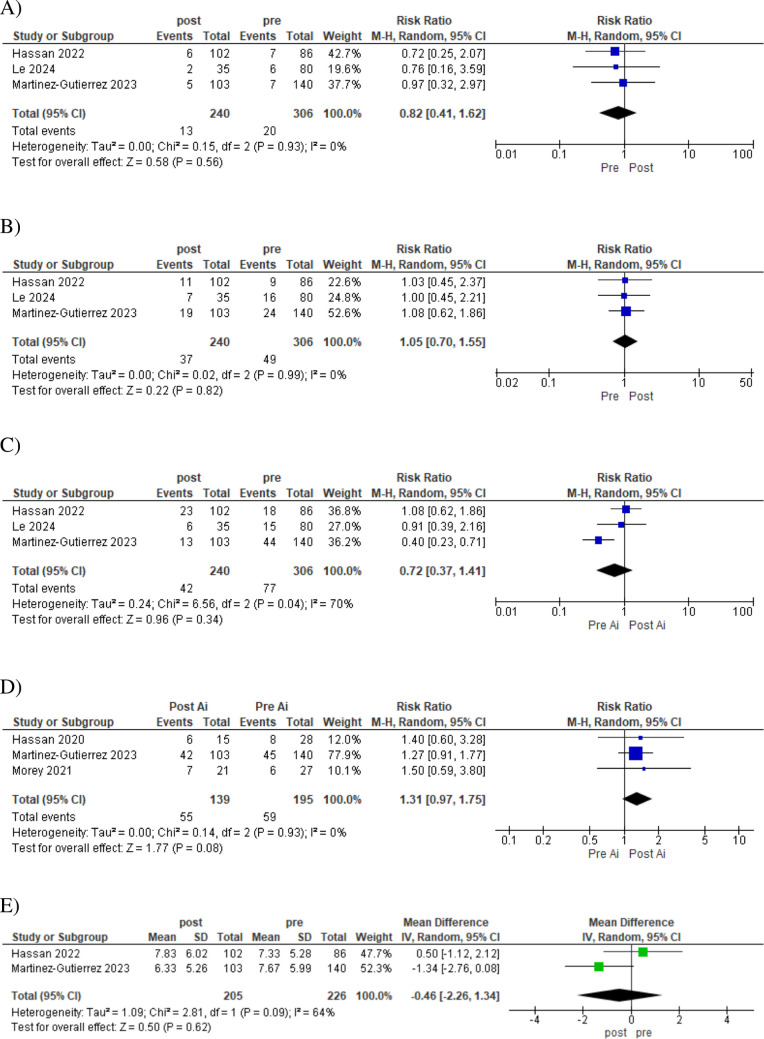
Forest plots of patient clinical outcomes pre- and post-implementation of Viz.ai software. **A** Symptomatic ICH, **B** any ICH, C mortality, **D** mRS score ≤ 2 at 90 days, **E** length of hospital stay

However, despite the workflow improvements and enhancement of significant results regarding the time-based outcomes, our analysis did not reveal statistically significant differences in certain clinical outcomes. The incidence of symptomatic intracranial hemorrhage (RR 0.82, 95% CI 0.41 to 1.62, *p* = 0.56) and any intracranial hemorrhage (RR 1.05, 95% CI 0.70 to 1.55, *p* = 0.82) remained comparable between the AI-assisted and control groups (Figure 4A and B). Similarly, mortality rates were not significantly different (RR 0.72, 95% CI 0.37 to 1.41, *p* = 0.34) (Figure 4C).

The proportion of patients achieving functional independence (modified Rankin Scale score ≤ 2) at 90 days showed a trend towards improvement, albeit not reaching statistical significance (RR 1.31, 95% CI 0.97 to 1.75, *p* = 0.08) (Figure 4D). These results warrant further investigation, as it hints at a potential translation of workflow efficiencies into improved patient outcomes. Lastly, we found no significant difference in the length of hospital stay between the two groups (RR −0.46, 95% CI −2.26 to 1.34, *p* = 0.62) (Figure 4E) suggesting that the observed workflow improvements did not necessarily translate into shorter hospitalizations.

## Discussion

The integration of AI in stroke care, particularly through platforms like VIZ.ai, has demonstrated significant potential in enhancing the detection of large vessel occlusion (LVO) and improving stroke workflow metrics in hospital settings.

Our systematic review and meta-analysis, encompassing 12 studies with a total of 15,595 patients, provide compelling evidence for the positive impact of AI integration on crucial time-based outcomes in stroke management. Among the interesting findings of our analysis is the substantial reduction in time from CT angiography (CTA) to the initiation of endovascular treatment (EVT). With a SMD of −0.71 (95% CI −0.98 to −0.44, *p* < 0.00001), this result underscores a marked acceleration in a critical phase of acute stroke care. This improvement aligns with the findings demonstrated by Murray et al. [1], in which they reported the superior performance of machine learning algorithms, particularly convolutional neural networks (CNNs), in detecting LVOs with a sensitivity of 85% compared to 68% for traditional methods [21].

The VIZ.ai platform has been noted for its high validation and application in emergency stroke systems, offering real-time LVO detection and activation of treatment protocols. The significant decrease in door-to-groin puncture (DTG) time (SMD −0.50, 95% CI −0.66 to −0.35, *p* < 0.00001) further emphasizes the efficiency gains brought about by AI integration. Those results are corroborated by Van Orden et al. in the VISIION-S study [40], which demonstrated a remarkable 39% reduction in door-to-groin times for off-hours LVO cases following the implementation of VIZ.ai [40]. The sustained nature of these improvements over a 17-month period highlights the long-term benefits of AI in optimizing stroke workflows. The shortened interval from CTA to successful recanalization (SMD −0.55, 95% CI −0.76 to −0.33, *p* < 0.00001) is another critical finding of our analysis. The estimated reduction in time to reperfusion is likely to have significant clinical implications, as faster recanalization has been associated with improved functional outcomes and reduced mortality in patients with LVO [10, 43, 44]. Chandrabhatla et al. review article has further supported the discussed results and evidence, highlighting the role of AI tools, as VIZ.ai has significantly reduced the time from scan acquisition to treatment initiation [45]. In the intersection and need of inter-hospital transfers, the significant reduction in door-in–door-out times (SMD −0.49, 95% CI −0.71 to −0.28, *p* < 0.00001) is particularly noteworthy. This improvement could be attributed to more efficient triage and decision-making processes facilitated by the platform. Figurelle et al.’s has provided important evidence on the implementation of VIZ.ai for streamlining communication in stroke centers, providing additional support for our analysis findings at this point, in which they have demonstrated a 30% improvement in overall communication efficiency during critical periods [27]. The marked reduction in time to neurointerventional radiologist (NIR) notification (SMD −0.87, 95% CI −0.90 to −0.83, *p* < 0.001) is a key factor contributing to overall workflow optimization. For this outcome, only two studies contributed data, with the Devlin 2024 study accounting for 99.6% of the weight. By performing sensitivity analysis and excluding the Devlin 2024 study to measure its impact on the overall effect estimate, the analysis left us with a single study, which also yielded statistical significance. These findings are also consistent with the broader improvements in clinical workflows as reported in the Chandrabhatla et al. review article in which they discussed the AI frameworks and stroke care (45), where AI tools were shown to significantly reduce the time from scan acquisition to treatment initiation [45]. The observed increase in the rate of EVT post-transfer to comprehensive stroke centers (CSCs) (RR 1.46, 95% CI 1.16 to 1.84, *p* = 0.001) is an intriguing finding that warrants further investigation. This increase could be attributed to improved patient selection and triage facilitated by AI.

While Viz.ai incorporates an artificial intelligence component in the form of a deep learning algorithm for automated LVO detection, it is best understood as a broader clinical workflow platform. The software facilitates real-time communication and automated alerting across multiple subspecialties through mobile and cloud-based tools. Therefore, the observed improvements in workflow metrics, such as reduced time to neurointerventional team notification, likely reflect both the performance of the AI algorithm and the enhanced coordination enabled by the overall software framework. It is important to recognize that while the deep learning model plays a central role in early identification, other features of the system, such as mobile notifications and automated image sharing, contribute substantially to the overall efficiency gains.

The comparative study by Delora et al. between VIZ.ai LVO and Rapid LVO software platforms offers insight into this improvement, highlighting Viz’s superior specificity (0.96 vs. 0.85) and positive predictive value (0.75 vs. 0.46) [46]. The lower rate of false positives with VIZ.ai likely contributes to more accurate patient selection for EVT, reducing unnecessary transfers and improving overall system efficiency. Despite the significant improvements in workflow metrics, our analysis did not reveal statistically significant differences in certain clinical outcomes, such as the incidence of intracranial hemorrhage or mortality rates. This underscores the complex nature of stroke outcomes and aligns with the cautionary note from Westwood et al.’s systematic review, which concludes that while AI demonstrates high sensitivity in LVO detection, no software currently available meets all the clinical needs for stroke triage [47]. The trend towards improved functional independence at 90 days (RR 1.31, 95% CI 0.97 to 1.75, *p* = 0.08), although not reaching statistical significance, is encouraging and warrants further investigation. This trend aligns with the cost-effectiveness model presented by Westwood et al. [47], which suggests that despite a marginal increase in costs for AI-guided diagnosis, the potential gains in quality-adjusted life years (QALYs) indicate long-term benefits, particularly in guiding mechanical thrombectomy [47].

The absence of a significant difference in the length of hospital stay between the AI-assisted and control groups is an interesting finding that merits further exploration. While improved workflow efficiency might be expected to reduce hospital stay duration, factors such as post-acute care arrangements, rehabilitation needs, and comorbidities may have a more substantial impact on the overall length of stay. Our findings must be interpreted in the context of certain limitations. The heterogeneity in study designs, AI implementation strategies, and outcome measures across the included studies may have influenced our results. This heterogeneity is reflected in the findings of Alwood et al., who identified protocol and scanner model variations as sources of discrepancies in AI performance across different centers [48]. Additionally, the relatively short follow-up periods in some studies may have limited our ability to detect long-term differences in clinical outcomes. The implications of our findings for clinical practice are substantial. The integration of AI-based tools like VIZ.ai into stroke care workflows has the potential to significantly reduce treatment delays and improve the efficiency of patient triage and management. However, as highlighted by Chandrabhatla et al., the importance of regulatory oversight in ensuring the safety and efficacy of AI algorithms cannot be overstated [45]. Healthcare systems considering the implementation of AI-based stroke triage systems should carefully evaluate the potential benefits in terms of workflow optimization against the costs and challenges of implementation. Future studies should consider reporting LVO locations with greater anatomical specificity to allow for more specified analysis of the effectiveness of AI-based detection tools like Viz.ai across different occlusion sites.

## Conclusion

Our results suggest that the implementation of the Viz.ai platform in stroke care holds significant potential for reducing EVT delays in patients with LVO and optimizing stroke flow metrics in CSC. However, additional studies evaluating patient’s clinical outcomes are needed to confirm its effectiveness in improving outcomes for individuals with LVO.

## Supplementary Information

Below is the link to the electronic supplementary material.

Supplementary file1 (DOCX 92 KB)

## Data Availability

No datasets were generated or analysed during the current study.
