# Supplementary material for: Automated Emergent Large Vessel Occlusion Detection Using Viz.ai Software and Its Impact on Stroke Workflow Metrics and Patient Outcomes in Stroke Centers: A Systematic Review and Meta-analysis
Source: Transl Stroke Res. 2025 May 8;16(6):2258–71. doi: 10.1007/s12975-025-01354-0 (PMC12596299; doi:10.1007/s12975-025-01354-0)
Supplement: Supplementary file 1 — Supplementary file1 (DOCX 92 KB) [file 12975_2025_1354_MOESM1_ESM.docx]

**Supplementary Online Content**

**Automated Emergent Large Vessel Occlusion Detection using Viz.ai Software and its Impact on Stroke Workflow Metrics and Patients Outcomes in Stroke Centers: A Systematic Review and Meta-analysis**

eAppendix 1. Sensitivity analysis for stroke workflow metrics outcomes

eAppendix 2. Funnel plots assessing publication bias for stroke workflow metrics outcomes

eAppendix 1. Sensitivity analysis for stroke workflow metrics outcomes


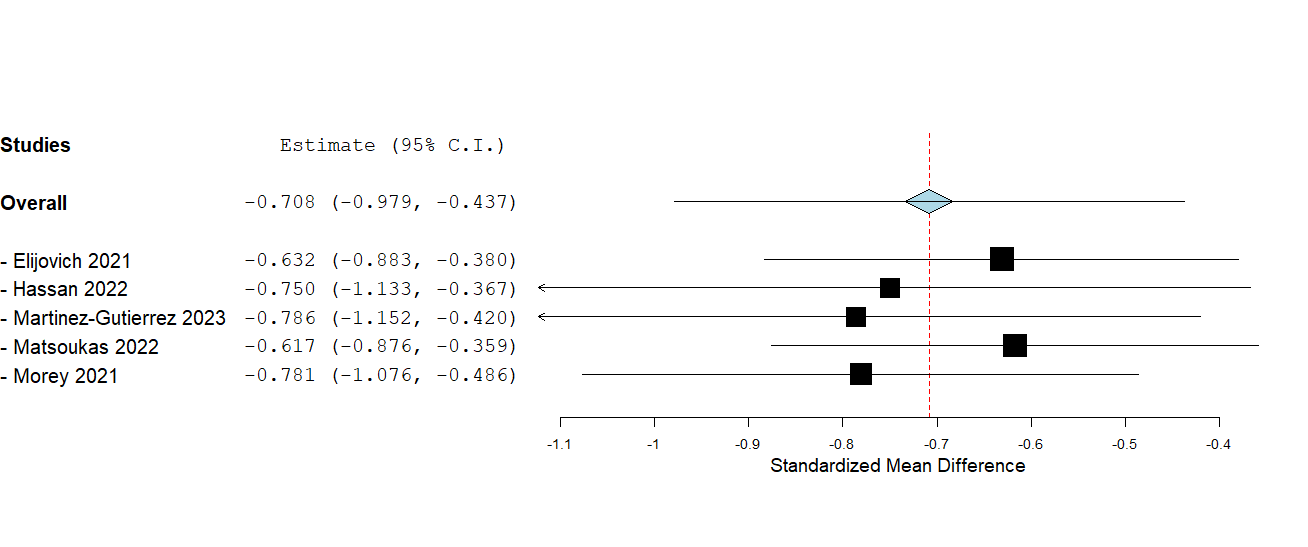


CTA to EVT start time


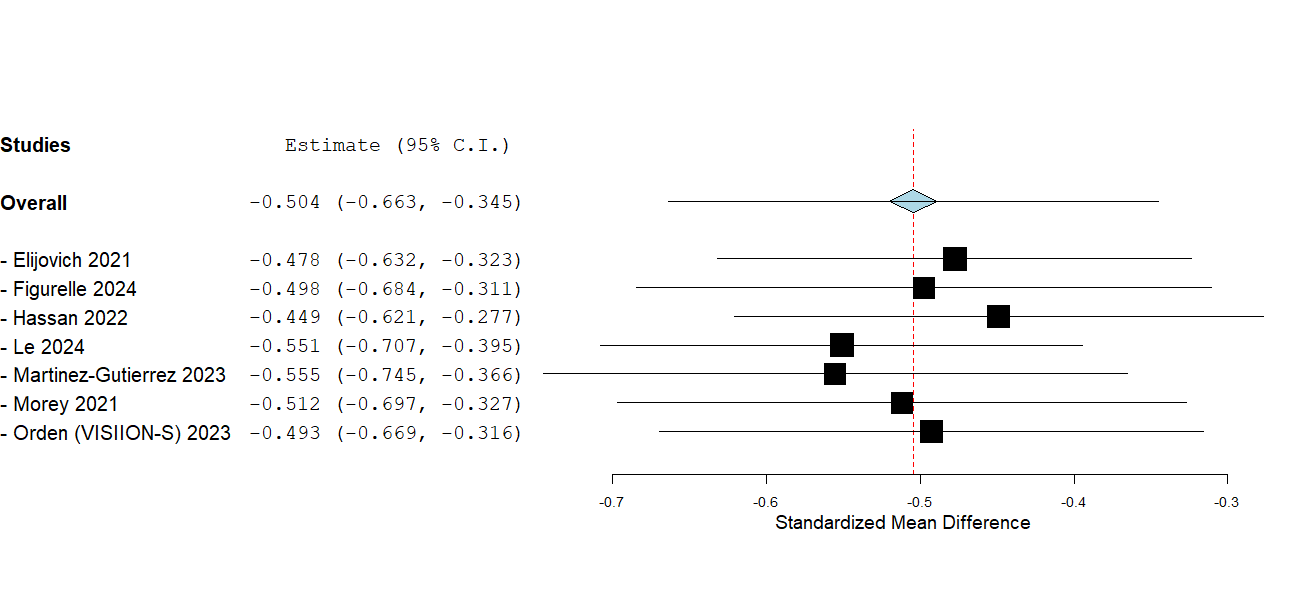


Door to groin puncture (DTG) time


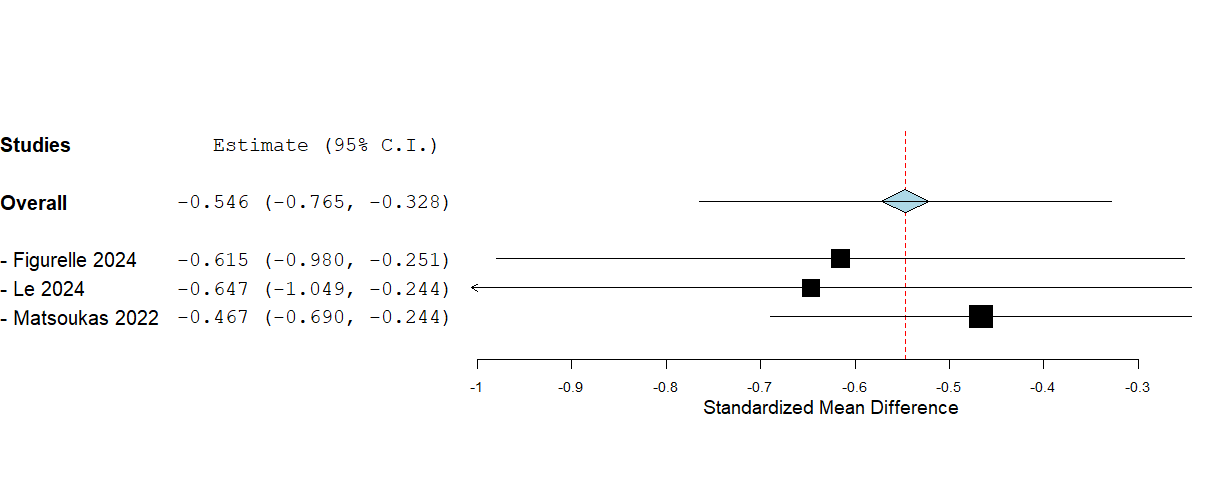


CTA to recanalization time


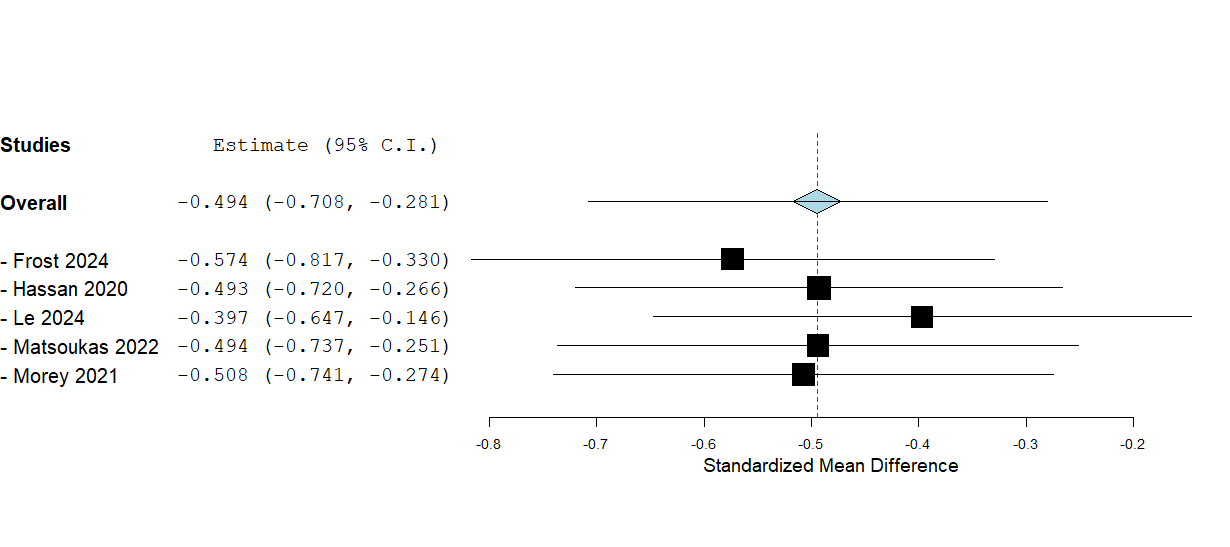


Door-in Door-out

eAppendix 2. Funnel plots assessing publication bias for stroke workflow metrics outcomes


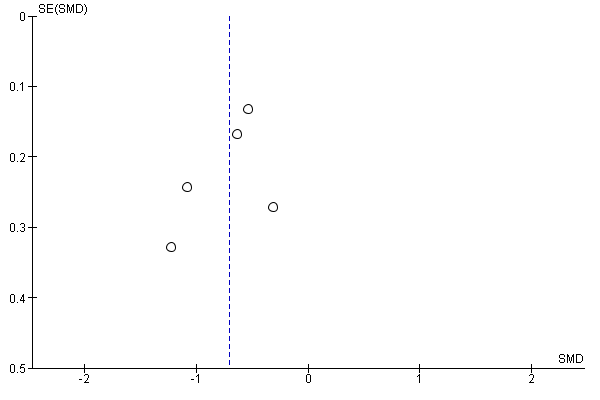


CTA to EVT start time


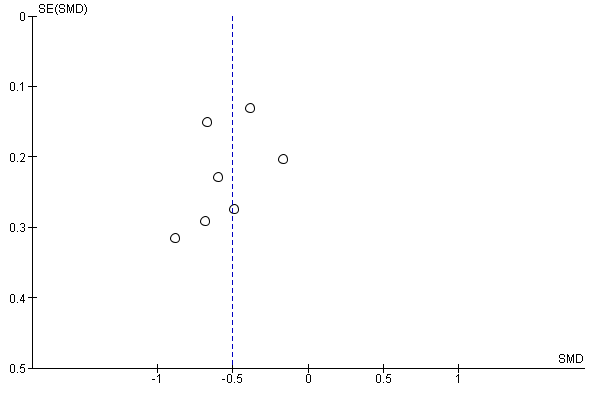


Door to groin puncture (DTG) time


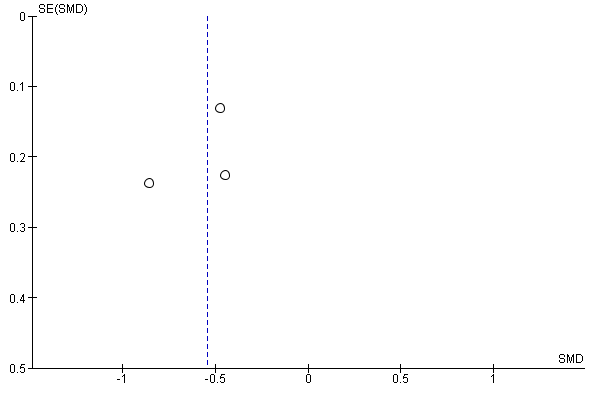


CTA to recanalization time


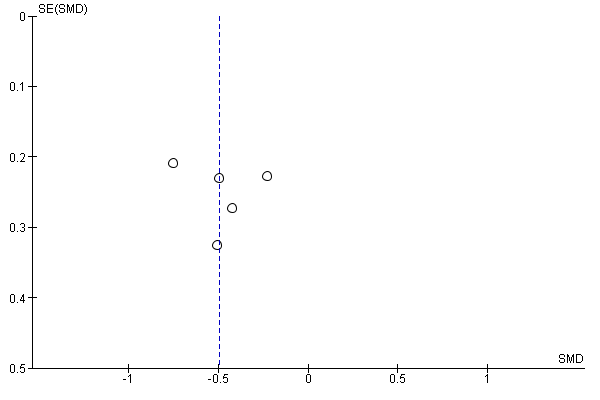


Door-in Door-out
